# Laparoscopic Fundoplication Using the Excluded Stomach Versus Conversion to Roux-en-Y Gastric Bypass for Refractory Reflux After One-Anastomosis Gastric Bypass: A Retrospective Comparative Analysis of Safety and 1-Year Outcomes

**DOI:** 10.3390/jcm15145717

**Published:** 2026-07-21

**Authors:** Antoine Soprani, Viola Zulian, Antonio Iannelli, Sergio Carandina

**Affiliations:** 1 Bariatric Surgery, Clinique Geoffroy-Saint Hilaire, 75005 Paris, France; antoinesoprani@gmail.com; 2 Bariatric and Metabolic Surgery, Clinique Saint Michel, ELSAN, 83100 Toulon, France; zulian.viola@gmail.com; 3 Bariatric and Metabolic Surgery, Clinique du Parc Impérial, 06000 Nice, France; chirurgiedigestive@priannelli.com

**Keywords:** reflux, one-anastomosis gastric bypass, Roux-en-Y gastric bypass, conversion, fundoplication, Reflux Symptom Index

## Abstract

**Background/Objectives**: Reflux remains one of the main limitations of one-anastomosis gastric bypass (OAGB), potentially impairing postoperative quality of life and requiring revisional surgery in refractory cases. Conversion to Roux-en-Y gastric bypass (RYGB) is currently considered the standard surgical treatment, although it involves additional anatomical modifications and potential morbidity. Fundoplication using the excluded stomach has recently emerged as a novel alternative approach. To compare the safety and efficacy of conversion to RYGB versus fundoplication using the excluded stomach in the management of refractory reflux after OAGB. Single-center retrospective study in a private bariatric surgery center. **Methods**: All consecutive patients who developed reflux refractory to optimized medical therapy after OAGB between January 2013 and January 2023 were included. The choice of revisional procedure—conversion to RYGB or laparoscopic fundoplication using the excluded stomach—evolved over time as the fundoplication technique was progressively introduced into clinical practice, reflecting a non-randomized, time-dependent allocation—a major limitation of this comparison. Primary outcomes were reflux resolution and postoperative complications. Secondary outcomes included reflux recurrence and the need for further surgical intervention. Reflux symptoms were assessed using the Reflux Symptom Index (RSI). **Results**: A total of 43 patients were included: 27 in the RYGB group and 16 in the fundoplication group. At 12 months, complete resolution of reflux symptoms was observed in 55.6% of patients in the RYGB group versus 93.7% in the fundoplication group (*p* = 0.02). Persistent reflux requiring reoperation occurred in 10 patients (37%) in the RYGB group, while no reoperations were required in the fundoplication group (*p* = 0.006). Late complications were significantly higher after RYGB conversion (25.9% vs. 0%, *p* = 0.035), mainly due to internal hernias. RSI scores significantly improved in both groups, with no significant difference in postoperative values. **Conclusions**: Fundoplication using the excluded stomach appears to be a feasible alternative to Roux-en-Y conversion for refractory reflux after OAGB, with favorable short-term outcomes. Given the non-randomized, time-dependent allocation, these findings are hypothesis-generating and require confirmation in larger, randomized studies.

## 1. Introduction

One-anastomosis gastric bypass (OAGB) has become one of the most widely performed bariatric procedures worldwide. Since the latest recommendations of the International Federation for the Surgery of Obesity and Metabolic Disorders (IFSO), the technique is no longer considered controversial and has demonstrated excellent outcomes in terms of weight loss, metabolic improvement, and overall safety [1]. Despite these advantages, bile reflux remains one of the main concerns associated with OAGB [2]. Although the true incidence varies among series, reflux after OAGB has been reported in approximately 5–10% of patients in earlier series, whereas more recent studies report rates approaching 30%, and it may significantly impair postoperative quality of life [3,4,5]. In addition to patient-related factors such as preexisting gastroesophageal reflux disease or hiatal hernia, several technical aspects have been implicated in its pathophysiology [6,7,8,9,10]. In an attempt to standardize the procedure, Mahawar et al. proposed key surgical principles including the creation of a long, narrow gastric pouch and a wide gastrojejunal anastomosis in order to establish a low-pressure system and reduce the likelihood of reflux [6]. The characterization of reflux following OAGB remains challenging. From a pathophysiological standpoint, the blind gastric pouch and the single gastrojejunal anastomosis create a low-resistance conduit through which biliopancreatic secretions can reflux and pool within the pouch, particularly when the pouch is long or the anastomosis is wide, increasing exposure of the gastric and esophageal mucosa to bile and pancreatic enzymes [11]. This alkaline, enzyme-rich refluxate appears more injurious to the esophageal mucosa than acid alone, and combined acid–biliary (mixed) reflux has been documented on impedance-pH monitoring in a substantial proportion of patients after OAGB [5]. Unlike classical gastroesophageal reflux disease, reflux after OAGB may be purely biliary or mixed with acid components. Using impedance monitoring, Doulami et al. demonstrated that mixed reflux is frequently observed in these patients, highlighting the complexity of its diagnosis and management [12]. In many cases, conservative management including dietary modifications and proton pump inhibitors can improve symptoms. However, a subset of patients develops severe and persistent reflux that becomes refractory to medical therapy. In these situations, surgical treatment may be required. The most commonly proposed surgical solution is conversion of OAGB to Roux-en-Y gastric bypass (RYGB), which diverts bile away from the gastric pouch and esophagus [13]. Although effective, this approach requires dismantling the original reconstruction and performing a more complex procedure. An alternative strategy consists of performing an antireflux procedure using the excluded stomach while preserving the OAGB anatomy. Fundoplication using the gastric remnant may theoretically restore the antireflux barrier without the need for conversion to RYGB [14]. However, data regarding the safety and effectiveness of this approach remain limited.

The aim of this retrospective study was therefore to compare the safety and efficacy of two surgical strategies for the management of refractory reflux after OAGB: conversion to Roux-en-Y gastric bypass versus fundoplication using the excluded stomach while preserving the initial OAGB configuration.

## 2. Materials and Methods

### 2.1. Study Design and Patient Selection

**Figure 1:**
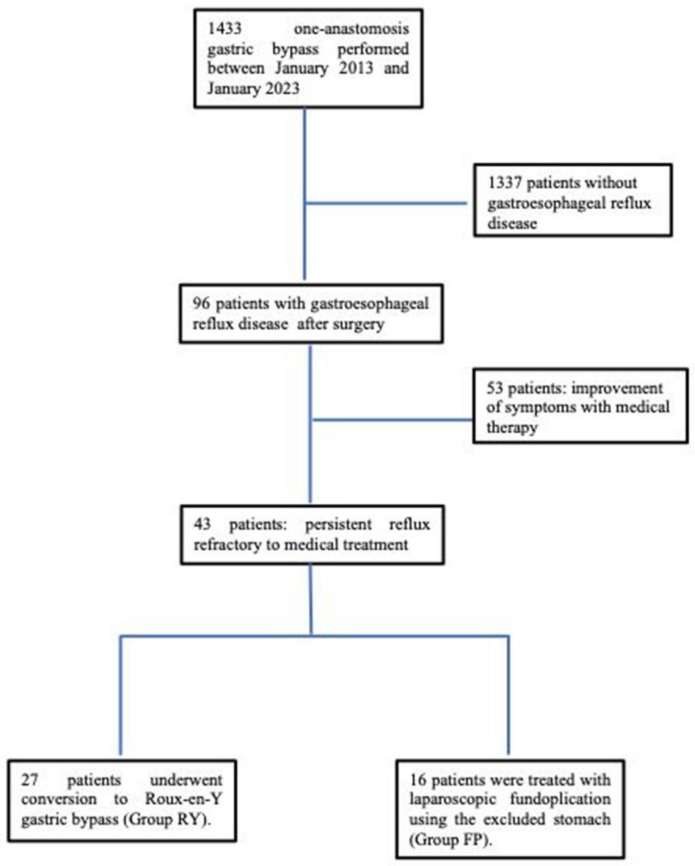
Study flow diagram.

In this retrospective study, we included all consecutive patients who underwent one-anastomosis gastric bypass (OAGB) between January 2013 and January 2023 at our private hospital. During this period, a total of 1433 OAGB procedures were performed. All index OAGB procedures were performed using a standardized omega-loop configuration with a biliopancreatic limb length of 200 cm. Among these patients, 96 (6.7%) developed symptomatic reflux after surgery, with a mean onset of 23.5 months (range 4–36 months). Reflux after OAGB was clinically characterized by heartburn, epigastric pain, nausea associated with anorexia, regurgitation, or vomiting of yellowish fluid suggestive of bile reflux. Given the lack of objective characterization by pH-impedance monitoring and/or Bilitec bile monitoring of the type of reflux (acid, biliary, or mixed) in our cohort, the term ‘reflux’ is used throughout this manuscript as the standard designation for the overall clinical and endoscopic entity studied. All patients were initially managed conservatively with proton pump inhibitors (PPIs) and dietary recommendations for at least 6 months. Fifty-three patients showed significant improvement of symptoms with medical therapy, whereas the remaining 43 patients (2.9% of the total cohort) presented persistent reflux refractory to medical treatment and were therefore referred for surgical management. These 43 patients constituted the study population. Among them, 27 patients underwent conversion to Roux-en-Y gastric bypass (RYGB) with preservation of the original OAGB gastrojejunal anastomosis, while the remaining 16 patients were treated with laparoscopic fundoplication using the excluded stomach (Figure 1).

### 2.2. Preoperative Assessment

According to French national recommendations, all patients underwent esophagogastroduodenoscopy (EGD) with systematic biopsies prior to revisional surgery. In the presence of *Helicobacter pylori* infection, eradication therapy was administered and confirmed with a breath test prior to surgery.

The preoperative endoscopic evaluation aimed to assess the presence and severity of esophagitis according to the Los Angeles classification, the presence of anastomotic ulcers or stenosis, the size of the gastric pouch, and the presence of esophageal bile reflux.

In addition, all patients underwent a computed tomography (CT) scan with oral contrast ingestion in order to detect hiatal hernia and to rule out technical issues related to the gastric pouch or the gastrojejunal anastomosis. All patients included in the study had a minimum follow-up of 12 months after revisional surgery. The clinical presence of reflux and its postoperative evolution were evaluated using the *Reflux Symptom Index (RSI)* score, a validated questionnaire composed of nine items scored from 0 (no symptom) to 5 (severe symptom). A score higher than 5 was considered indicative of significant reflux symptoms [15].

### 2.3. Outcomes

The primary outcomes of the study were: resolution of preoperative reflux symptoms and postoperative complication rate. Complications were classified as early (within the first postoperative month) or late (after the first postoperative month). Their severity was graded according to the Clavien–Dindo classification [16]. The secondary outcome was the recurrence of reflux symptoms and the need for additional treatment.

### 2.4. Surgical Technique

All procedures were performed laparoscopically. Conversions to Roux-en-Y gastric bypass were performed by two surgeons during an earlier period of the study, whereas laparoscopic fundoplication using the excluded stomach was performed by a single surgeon.

### 2.5. Conversion to Roux-en-Y Gastric Bypass

**Figure 2:**
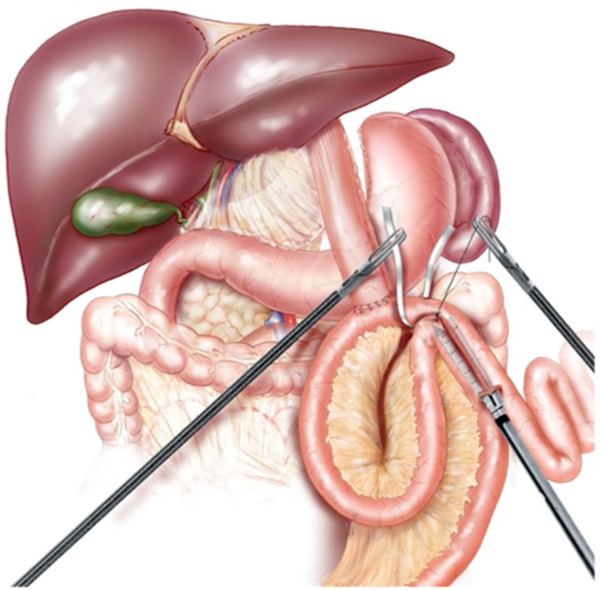
A new jejunojejunal anastomosis was created between the biliary limb adjacent to the gastrojejunal anastomosis and the efferent limb at a distance of approximately 70 cm from the gastrojejunal anastomosis.

**Figure 3:**
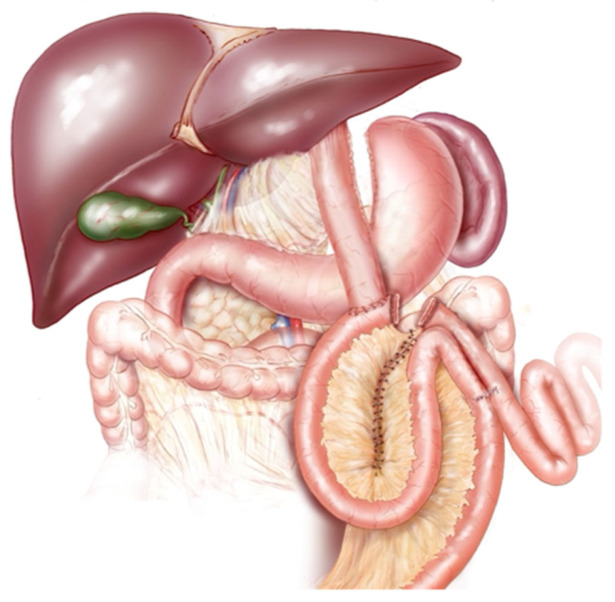
The biliary limb was subsequently divided between the two anastomoses in order to separate the alimentary and biliary limbs, and the mesenteric defects were closed.

After exploration of the abdominal cavity, the biliary limb was identified and its length measured. A jejunojejunal anastomosis was then created between the biliary limb adjacent to the gastrojejunal anastomosis and the efferent limb at a distance of approximately 70 cm from the gastrojejunal anastomosis (Figure 2). The biliary limb was subsequently divided between the two anastomoses using a linear stapler in order to separate the alimentary and biliary limbs. The procedure was completed by closure of the mesenteric defects, including Petersen’s space and the jejunojejunal mesenteric defect (Figure 3). Hiatal dissection and crural repair were not performed as part of this procedure, regardless of the presence of a hiatal hernia on preoperative imaging.

### 2.6. Fundoplication Using the Excluded Stomach

**Figure 4:**
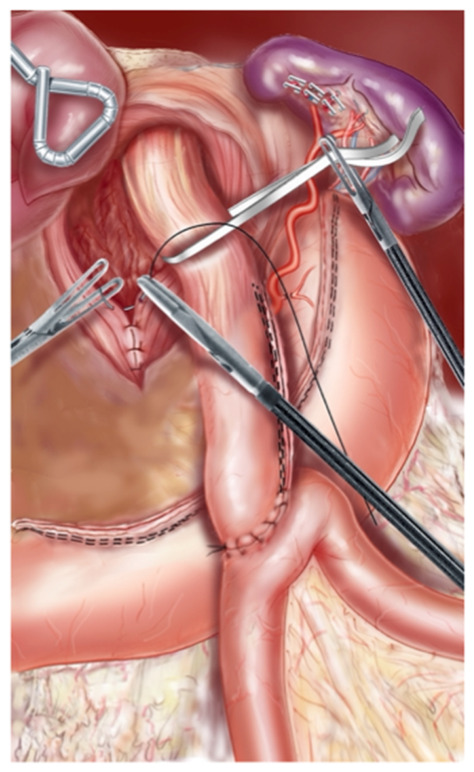
After complete mobilization of the gastric tube, the lower esophagus was dissected to reduce a potential hiatal hernia and a cruroplasty was performed.

**Figure 5:**
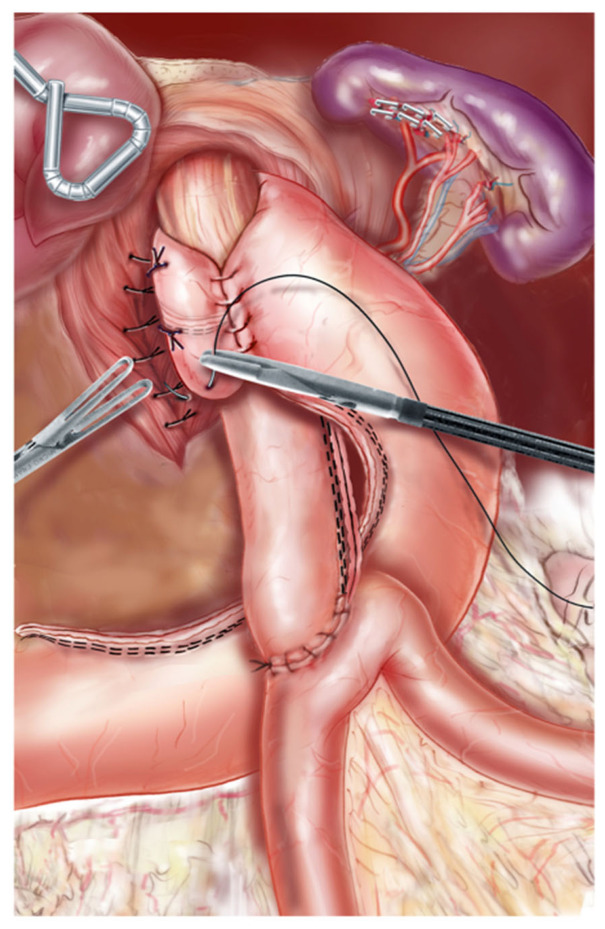
A 360° Nissen fundoplication measuring approximately 2.5–3 cm in length was constructed using the fundus of the excluded stomach, which was passed behind the esophagus through the retroesophageal space.

The first step consisted of adhesiolysis between the gastric pouch, the liver, and the excluded stomach. After complete mobilization of the gastric tube, the lower esophagus was dissected to reduce a potential hiatal hernia and a cruroplasty was performed (Figure 4). Hiatal hernia was treated by opening the lesser omentum and the phrenoesophageal membrane and by complete dissection of the diaphragmatic crura. After reduction of the hernial contents, the hernia sac was resected and the abdominal esophagus was carefully mobilized while preserving the vagus nerves and the left gastric vessels. The hiatus was closed using two to three interrupted non-absorbable sutures. In patients with a history of adjustable gastric banding (AGB), the residual fibrous capsule was systematically resected. A 360° Nissen fundoplication measuring approximately 2.5–3 cm in length was then constructed using the fundus of the excluded stomach, which was passed behind the esophagus through the retroesophageal space. To reduce the risk of postoperative dysphagia, extensive mobilization of the fundus was performed, including systematic division of the posterior gastric artery and the last two short gastric vessels (Figure 5).

### 2.7. Postoperative Management and Follow-Up

An upper gastrointestinal contrast study was performed on postoperative day (POD) 1. In the absence of leakage, oral feeding was started according to the nutritional protocol. Patients were discharged on POD 1 following clinical evaluation and confirmation of adequate oral intake. All patients received multivitamin supplementation until the 3-month follow-up re-evaluation, proton pump inhibitor therapy (40 mg/day) during the first postoperative month, and anticoagulant prophylaxis for one month. Follow-up visits were scheduled at 1, 3, 6, 9, and 12 months, and twice yearly thereafter. At 12 months postoperatively, the RSI questionnaire was administered. Upper gastrointestinal contrast studies were scheduled at 1 and 3 years, while endoscopic evaluation was planned at 5 years.

### 2.8. Statistical Analysis

Descriptive analyses were performed according to the nature of the variables. Categorical variables were expressed as absolute numbers and percentages. Comparisons between categorical variables were performed using the Chi-square test or Fisher’s exact test when appropriate. Continuous variables were expressed as mean ± standard deviation, median, and range. Comparisons between continuous variables were performed using Student’s *t*-test or the Mann–Whitney U test depending on data distribution. The confidence interval of the revisional surgery rate for persistent reflux was calculated using the exact Clopper–Pearson method. For 2 × 2 tables containing at least one zero-event cell (Tables 2 and 3), odds ratios and their 95% confidence intervals were calculated after applying the Haldane–Anscombe continuity correction (adding 0.5 to each cell), with confidence intervals derived using Woolf’s method on the log-odds scale. All statistical tests were two-sided, and a *p* value < 0.05 was considered statistically significant. Statistical analyses were performed using SAS software (version 9.4; SAS Institute Inc., Cary, NC, USA).

## 3. Results

### 3.1. Patient Characteristics

A total of 43 consecutive patients with gastroesophageal reflux refractory to medical treatment after OAGB were included in the study. Among them, 27 patients underwent conversion to Roux-en-Y gastric bypass with preservation of the original gastrojejunal anastomosis (RY group), while 16 patients underwent laparoscopic fundoplication using the excluded stomach (FP group). There were no statistically significant differences between the two groups in terms of age at revisional surgery, although patients in the FP group were slightly older (44.5 ± 10 years in the RY group vs. 50.8 ± 12.9 years in the FP group; *p* = 0.13), or in terms of preoperative BMI (27 ± 4.7 kg/m^2^ in the RY group vs. 29 ± 3.2 kg/m^2^ in the FP group, *p* = 0.13). The interval between primary OAGB and revisional surgery was significantly longer in the FP group compared to the RY group (81.9 ± 49.7 months vs. 39.6 ± 32.6 months; *p* = 0.0051). All patients presented with a hiatal hernia on both EGD and CT scan. A history of previous restrictive surgery was more frequent in the RY group, with 15 patients (55.5%) having undergone adjustable gastric banding (AGB), including two patients later converted to sleeve gastrectomy, and one additional patient with primary sleeve gastrectomy, while only six patients (60%) in the FP group had a history of AGB.

Preoperative endoscopy revealed esophagitis in the majority of patients. In the RY group, esophagitis was classified as Los Angeles grade C in 2 patients, grade B in 8 patients, and grade A in 7 patients, while 10 patients had normal endoscopic findings. In the FP group, esophagitis was grade C in 1 patient, grade B in 5 patients, and grade A in 6 patients, while 4 patients had no esophagitis. Bile was detected in the gastric pouch in all patients, with evidence of esophageal bile reflux in 7 patients in the RY group and 3 patients in the FP group. No anastomotic stenosis or marginal ulcers were observed. Histological analysis consistently showed foveolar hyperplasia. The mean preoperative RSI score was 7.5 ± 1 in the RY group and 7.6 ± 1.2 in the FP group (*p* = 0.76). The mean follow-up duration was 5.4 ± 2.6 years in the RY group and 2.8 ± 1.8 years in the FP group (*p* = 0.005) (Table 1).

### 3.2. Operative Outcomes

All procedures were completed laparoscopically except for one case in the RY group, which required conversion to open surgery due to vascular injury during trocar insertion. The mean operative time was significantly shorter in the RY group compared to the FP group (46.8 ± 6.7 min vs. 88 ± 14.8 min; *p* < 0.0001). In the FP group, operative time was significantly longer in patients with a history of AGB (101.5 ± 5.1 min vs. 79.9 ± 12.5 min; *p* = 0.001) (Table 2).

### 3.3. Postoperative Complications

Early postoperative complications (<30 days) occurred in 1 patient (3.7%) in the RY group and in 1 patient (6.25%) in the FP group (*p* = 1). In the RY group, one patient required reoperation on postoperative day 7 for a trocar-site Richter’s hernia (Clavien–Dindo IIIb). In the FP group, one patient developed biliary peritonitis due to gastrojejunal injury requiring reoperation on postoperative day 1 (Clavien–Dindo IV). No mortality occurred.

Late complications (>30 days) were significantly more frequent in the RY group. In the RY group, 5 patients developed internal hernias requiring surgical repair (3 Petersen hernias and 2 jejunojejunal mesenteric hernias). One patient developed severe malnutrition requiring reversal to normal anatomy, and one patient presented a bleeding marginal ulcer with stenosis requiring redo gastrojejunal anastomosis. Overall, late complications occurred in 7 patients (25.9%). No late complications were observed in the FP group (*p* = 0.035; OR: 12.07; 95% CI [0.6413–227.2897]), again reflecting the small number of events and limited precision (Table 2).

### 3.4. Reflux Outcomes

At 12-month follow-up, complete resolution of reflux symptoms was observed in 15 patients (55.6%) in the RY group and in 15 patients (93.7%) in the FP group (*p* = 0.02; OR 0.14, 95% CI [0.0225–0.9086]). Partial improvement of symptoms with reduced PPI use was observed in 2 patients in the RY group and in 1 patient in the FP group. Persistent severe reflux requiring further surgery occurred in 10 patients in the RY group, while no patients in the FP group required reoperation (*p* = 0.006; OR 19.8, 95% CI [1.07–365.5]). Given the small number of events, this estimate is imprecise and should be interpreted with caution. Among the patients reoperated, 5 patients underwent fundoplication on the RYGB, while 5 patients required gastrectomy with pouch resizing and redo gastrojejunal anastomosis. Notably, 70% of these patients had a history of AGB.

The mean RSI score significantly improved in both groups. In the RY group, the RSI decreased from 7.5 ± 1 preoperatively to 2.8 ± 3.2 postoperatively (*p* < 0.0001). In the FP group, it decreased from 7.6 ± 1.2 to 1.1 ± 1.2 (*p* < 0.0001). There was no statistically significant difference between the two groups in postoperative RSI scores (*p* = 0.37). However, the rate of persistent reflux was significantly higher in the RY group (44.4%) compared to the FP group (6.25%) (*p* = 0.01; OR 8.33, 95% CI [1.33–52.16]) (Table 3).

## 4. Discussion

In the present study, we compared two surgical strategies for the management of refractory reflux following OAGB: conversion to Roux-en-Y gastric bypass and fundoplication using the excluded stomach. Our results suggest that fundoplication is a safe and effective alternative, providing excellent symptom control with a lower rate of postoperative complications. In particular, fundoplication appeared to achieve a higher rate of reflux resolution while avoiding the need for additional intestinal reconstruction and its associated risks.

The pathophysiology of reflux after OAGB remains multifactorial and not yet fully understood. In our series, all patients presented with a hiatal hernia, highlighting its central role in the development of postoperative reflux. Hiatal hernia is known to impair the anti-reflux barrier by altering the function of the lower esophageal sphincter, thereby facilitating the retrograde flow of gastric and/or biliary contents into the esophagus. Winstanley et al., in a series of 63 patients with HH and operated on OAGB, found an impressive 20.6% of patients with a postoperative de novo GERD and who required long-term PPI treatment [17]. Another important factor is the history of previous restrictive procedures, particularly adjustable gastric banding (AGB), which was present in more than half of our patients. Several studies have identified AGB as a significant risk factor for postoperative reflux after OAGB, likely due to long-term alterations of the esophagogastric junction, including fibrosis, motility disorders, and sphincter dysfunction [18,19,20]. It should be noted that AGB procedures were performed years earlier, often by different surgical teams, at a time when AGB was a widely used first-line procedure and hiatal hernia was not systematically considered a contraindication. The association between prior AGB and increased reflux risk after OAGB has only been established in more recent literature. A similar pathophysiological mechanism has also been described after sleeve gastrectomy. The creation of a long and narrow gastric tube, combined with disruption of the phreno-esophageal ligament and partial section of the sling fibers, weakens the anti-reflux barrier and may promote both hiatal hernia formation and reflux [21,22]. In this regard, OAGB shares important anatomical and functional similarities with sleeve gastrectomy, which may explain the occurrence of reflux in both procedures.

Recently, fundoplication using the excluded stomach has been proposed as a novel approach for the management of refractory reflux after OAGB. In the series reported by Werapitiya et al., this technique demonstrated promising short-term results with significant improvement in reflux-related quality of life scores and high patient satisfaction [14]. Our results are consistent with these findings and further strengthen the evidence supporting this approach. However, in contrast to previously published series with limited sample size, our study provides a direct comparison with the current standard surgical treatment, allowing for a more comprehensive evaluation of its relative benefits and risks. Indeed, conversion to Roux-en-Y gastric bypass has long been considered the gold standard for managing refractory reflux after OAGB [23]. This approach effectively diverts bile away from the gastric pouch and esophagus. However, it does not directly address the underlying dysfunction of the esophagogastric junction. For this reason, particularly in patients with mixed reflux, GERD may persist despite conversion [20]. In the series by Winstanley et al., more than 60% of patients converted to RYGB for severe GERD had persistent reflux of varying severity [17]. This persistent GERD could be explained by the creation of a too-long gastric pouch and a deficient inferior esophageal sphincter, frequently favored by a previous gastric banding history [24]. Moreover, conversion to RYGB introduces new anatomical modifications, including the creation of mesenteric defects, which significantly increases the risk of internal hernia [25]. This was clearly reflected in our series, where a substantial proportion of patients in the RY group required further surgical intervention for internal hernia. In addition, RYGB conversion may expose patients to long-term complications such as malabsorption, marginal ulcers, and nutritional deficiencies, particularly in cases where the initial biliopancreatic limb was long [26]. These aspects should be carefully considered when proposing revisional surgery in patients who have already achieved satisfactory weight loss. In contrast, fundoplication using the excluded stomach offers a more physiological approach by trying to restore the competence of the lower esophageal sphincter without altering the intestinal anatomy. The creation of the fundoplication wrap allows both maintenance of the distal esophagus in its intra-abdominal position and restoration of a physiological pressure gradient between the esophagus and the gastric pouch [27]. The 360-degree wrap acts as an external control mechanism around the gastric pouch, increasing the pressure at the level of the cardia and thereby improving its competence while reducing reflux. However, hiatal hernia repair, performed concomitantly in all FP patients but not in the RY group, may also contribute independently to this effect, and the relative contribution of crural closure versus the fundoplication wrap cannot be disentangled in the present study. Additionally, this approach avoids the creation of new anastomoses and mesenteric defects, thereby reducing the risk of internal hernia and other complications related to intestinal reconstruction. The main drawback of this technique is its technical complexity, particularly in patients with a history of AGB, where dense posterior adhesions may increase operative difficulty and duration. Nevertheless, in experienced hands, the procedure appears safe and reproducible.

This study has several limitations. The most important is the non-randomized, time-dependent patient allocation: the choice of procedure was not based on predefined criteria but evolved as fundoplication was progressively introduced into clinical practice. Early patients were managed by RYGB conversion, while fundoplication was proposed subsequently as surgical experience accumulated. As a result, the two groups largely correspond to different historical periods. This is reflected in the significantly longer interval between primary OAGB and revisional surgery in the FP group (81.9 ± 49.7 vs. 39.6 ± 32.6 months; *p* = 0.005) and in the shorter postoperative follow-up available for this group (2.8 ± 1.8 vs. 5.4 ± 2.6 years; *p* = 0.005). This temporal separation implies a potential learning-curve effect: surgical experience and perioperative management improved over time and may have influenced outcomes independently of the procedure. Furthermore, RYGB conversions were performed by two surgeons during an earlier period, whereas all fundoplications were performed by a single surgeon, introducing surgeon-related variability that cannot be disentangled from the comparison. For these reasons, the results should be regarded as hypothesis-generating rather than demonstrative of causal superiority. An additional structural confounder is that hiatal hernia repair was performed in all FP patients as part of the fundoplication procedure, but not systematically in the RY group. Since hiatal closure is a prerequisite for constructing the wrap, this component cannot be dissociated from the fundoplication technique itself, and part of the superior reflux control observed in the FP group may be attributable to hiatal reconstruction rather than to the fundoplication wrap alone. Sample size remains relatively small, particularly in the fundoplication group, limiting the statistical power of the analysis, resulting in wide confidence intervals around key estimates, which should be interpreted as indicative rather than precise. Follow-up was also significantly shorter in the FP group (2.8 ± 1.8 vs. 5.4 ± 2.6 years), limiting the detection of late complications and reflux recurrence. Although most reflux-related failures occur within the first postoperative year—the time frame of our primary endpoint—longer follow-up may reveal additional events not yet apparent. Reflux diagnosis was based on clinical symptoms and RSI score, without objective functional tests such as pH-impedance monitoring, Bilitec bile monitoring, esophageal manometry, or scintigraphy. Accordingly, this study evaluates symptom response to revisional surgery rather than objectively confirmed reflux. Finally, preoperative endoscopic and radiological data from the time of the index bariatric procedure were unavailable for many patients—particularly those with a prior history of adjustable gastric banding performed by different surgical teams—precluding retrospective assessment of pre-existing hiatal hernia or reflux. Finally, this is a single-center study performed by experienced surgeons, which may limit the generalizability of the results.

## 5. Conclusions

In conclusion, within the limits of a non-randomized comparison subject to temporal and selection bias, fundoplication using the excluded stomach appears to be a feasible and promising alternative to Roux-en-Y conversion for refractory reflux after OAGB. In this preliminary series, it was associated with favorable symptom control while preserving the original intestinal anatomy, thereby avoiding the morbidity of intestinal reconstruction—including the risk of internal hernia—which proved to be the principal driver of late complications in the RYGB group. The lower reoperation rate for persistent reflux observed in the fundoplication group is encouraging, but requires confirmation in larger, prospective, and ideally randomized cohorts before influencing clinical practice. It should also be noted that, in the absence of objective functional assessment—including pH-impedance monitoring, Bilitec bile monitoring, and esophageal manometry—the reported outcomes reflect symptom improvement as evaluated by the RSI score, rather than objectively confirmed reflux control. Fundoplication using the excluded stomach may be especially worth investigating in patients with evidence of esophagogastric junction dysfunction, associated hiatal hernia, or a history of adjustable gastric banding, where addressing the anatomical substrate of reflux appears more clinically relevant than diverting the biliary limb alone. Further prospective, multicenter studies with longer follow-up are warranted to confirm these findings, define the optimal patient selection criteria, and evaluate the long-term durability of the fundoplication wrap in this anatomical context.

## Figures and Tables

**Table 1:** Baseline patient characteristics.

Demographics & Anthropometrics	RY Group	FP Group	*p*
Age at revision (years), mean ± SD	44.5 ± 10	50.8 ± 12.9	0.13
Sex ratio (F/M)	22/5	13/3	1
BMI at revision (kg/m^2^), mean ± SD	27.0 ± 4.7	29 ± 3.2	0.13
Interval OAGB → revision (months), mean ± SD	39.6 ± 32.6	81.9 ± 49.7	0.005
Follow-up after revision (years), mean ± SD	5.4 ± 2.6	2.8 ± 1.8	0.005
**Surgical history, *n* (%)**			
History of AGB	15 (55.5%)	6 (37.5%)	0.34
AGB → sleeve conversion	2 (7.4%)	0 (0%)	/
Primary sleeve gastrectomy	1 (3.7%)	0 (0%)	/
**Preoperative endoscopic findings, *n* (%)**			
Hiatal hernia	27 (100%)	16 (100%)	1
Esophagitis—LA grade C	2 (7.4%)	1 (6.3%)	1
Esophagitis—LA grade B	8 (29.6%)	5 (31.3%)	1
Esophagitis—LA grade A	7 (25.9%)	6 (37.5%)	0.5
No esophagitis	10 (37.0%)	4 (25.0%)	0.51
Esophageal bile reflux	7 (25.9%)	3 (18.8%)	0.71
Foveolar hyperplasia (histology)	27 (100%)	16 (100%)	1
**Preoperative symptom score**			
RSI score, mean ± SD	7.5 ± 1.0	7.6 ± 1.2	0.76

AGB: adjustable gastric banding; BMI: body mass index; FP: fundoplication using the excluded stomach; LA: Los Angeles classification; OAGB: one-anastomosis gastric bypass; RSI: Reflux Symptom Index; RY: conversion to Roux-en-Y gastric bypass; SD: standard deviation.

**Table 2:** Operative and postoperative outcomes.

Operative Outcomes	RY Group	FP Group	*p*
Operative time (min), mean ± SD	46.8 ± 6.7	88.0 ± 14.8	<0.0001
Conversion to open surgery, *n* (%)	1 (3.7%)	0 (0%)	1
**Early complications (<30 days), *n* (%)**			
Any early complication	1 (3.7%)	1 (6.3%)	1.00
Trocar-site Richter hernia (CDIIIb)	1 (3.7%)	0 (0%)	1
Biliary peritonitis (CDIV)	0 (0%)	1 (6.3%)	1
30-day mortality	0 (0%)	0 (0%)	1
**Late complications (>30 days), *n* (%)**			
Any late complication	7 (25.9%)	0 (0%)	0.035
Internal hernia	5 (18.5%)	0 (0%)	0.13
Petersen’s hernia	3 (11.1%)	0 (0%)	0.28
Jejunojejunal mesenteric hernia	2 (7.4%)	0 (0%)	0.52
Severe malnutrition	1 (3.7%)	0 (0%)	1
Marginal ulcer with stenosis	1 (3.7%)	0 (0%)	1

CD: Clavien–Dindo classification; FP: fundoplication using the excluded stomach; GJ: gastrojejunal; NS: not significant; RY: conversion to Roux-en-Y gastric bypass.

**Table 3:** Reflux outcomes at 12-month follow-up.

Reflux Outcomes at 12 Months, *n* (%)				
Complete resolution of reflux symptoms	15 (55.6%)	15 (93.7%)	0.02	0.14 [0.02–0.91]
Partial improvement (reduced PPI use)	2 (7.4%)	1 (6.3%)	1	1.01 [0.12–8.44]
Persistent reflux (any)	10 (44.4%)	1 (6.3%)	0.01	8.33 [1.33–52.2]
Reoperation for persistent reflux	10 (37.0%)	0 (0%)	0.006	19.8 [1.07–365]
Fundoplication on RYGB	5 (18.5%)	—	—	—
Pouch resize + redo GJ anastomosis	5 (18.5%)	—	—	—
**Reflux Symptom Index (RSI) scores, mean ± SD**				
Preoperative RSI	7.5 ± 1.0	7.6 ± 1.2	*p* = 0.76	—
Postoperative RSI	2.8 ± 3.2	1.1 ± 1.2	*p* = 0.37	—
Within-group improvement	<0.0001	<0.0001	*p*—	—

CI: confidence interval; FP: fundoplication using the excluded stomach; OR: odds ratio; PPI: proton pump inhibitor; RSI: Reflux Symptom Index; RY: conversion to Roux-en-Y gastric bypass; RYGB: Roux-en-Y gastric bypass.

## Data Availability

The original contributions presented in this study are included in the article. Further inquiries can be directed to the corresponding author.

## References

[1] De Luca M., Tie T., Ooi G., Higa K., Himpens J., Carbajo M.-A., Mahawar K., Shikora S., Brown W.A. (2018). Mini Gastric Bypass—One Anastomosis Gastric Bypass (MGB-OAGB) IFSO Position Statement. Obes. Surg..

[2] Mahawar K.K., Car W.R., Balupuri S., Smal P.K. (2014). Controversy surrounding “mini” gastric bypass. Obes. Surg..

[3] Carandina S., Soprani A., Zulian V., Cady J. (2021). Long-term results of one anastomosis gastric bypass: A single center experience with a minimum follow-up of 10 years. Obes. Surg..

[4] Ghiassi S., Nimeri A., Aleassa E.M., Grover B.T., Eisenberg D., Carter J., American Society for Metabolic and Bariatric Surgery Clinical Issues Committee (2024). American Society for Metabolic and Bariatric Surgery position statement on one-anastomosis gastric bypass. Surg. Obes. Relat. Dis..

[5] Felsenreich D.M., Vock N., Zach M.L., Kristo I., Jedamzik J., Bichler C., Eichelter J., Mairinger M., Gensthaler L., Nixdorf L. (2025). Update on esophageal function, acid and non-acid reflux after one-anastomosis gastric bypass (OAGB): High-resolution manometry, impedance-24-h pH-metry, and gastroscopy in a prospective mid-term study. Surg. Endosc..

[6] Mahawar K.K., Parmar C., Graham Y. (2019). One anastomosis gastric bypass: Key technical features, and prevention and management of procedure-specific complications. Minerva Chir..

[7] Musella M., Susa A., Manno E., De Luca M., Greco F., Raffaelli M., Cristiano S., Milone M., Bianco P., Vilardi A. (2017). Complication following the Mini/One Anastomosis Gastric Bypass (MGB/OAGB): A multi-institutional Survey on 2678 Patients with a Mid-Term (5 years) Follow up. Obes. Surg..

[8] Apers J., Wijkmans R., Totte E., Emous M. (2018). Implementation of Mini Gastric bypass in the Netherlands: Early and midterm results from high-volume unit. Surg. Endosc..

[9] Tolone S., Cristiano S., Savarino E., Lucido S.F., Fico D.I., Docimo L. (2016). Effects of Omega-Loop Bypass on esophagogastric junction function. Surg. Obes. Relat. Dis..

[10] Saarinen T., Räsänen J., Salo J., Loimaala A., Pitkonen M., Leivonen M., Juuti A. (2017). Bile Reflux Scintigraphy after Mini-Gastric Bypass. Obes. Surg..

[11] Márquez M., García-Redondo M., Maturana-Ibáñez V., Estébanez-Ferrero B., Fernández-Alonso A., Rubio-Gil F., Zamora Soler J.A., Ferrer-Ayza M. (2023). Bile reflux and marginal ulcers after one-anastomosis gastric bypass (OAGB). A narrative review. Cir. Esp..

[12] Doulami G., Triantafyllou S., Albanopoulos K., Natoudi M., Zografos G., Theodorou D. (2018). Acid and nonacid gastroesophageal reflux after single anastomosis gastric bypass. An objective assessment using 24-hour multichannel intraluminal impedance-pH metry. Surg. Obes. Relat. Dis..

[13] Ramos A.C., Chevallier J.M., Mahawar K., Brown W., Kow L., White K.P., Shikora S. (2020). IFSO (International Federation for Surgery of Obesity and Metabolic Disorders) consensus conference statement on one-anastomosis gastric bypass (OAGB-MGB): Results of a modified Delphi study. Obes. Surg..

[14] Werapitiya S.B., Ruwanpura S.P., Coulson T.R. (2022). Laparoscopic fundoplication using the excluded stomach as a novel management option for refractory bile reflux following one anastomosis gastric bypass (OAGB). Obes. Surg..

[15] Abraham Z.S., Kahing A.A. (2022). Utility of reflux finding score and reflux symptom index in diagnosis of laryngopharyngeal reflux disease. Laryngoscope Investig. Otolaryngol..

[16] Clavien P.A., Barkun J., de Oliveira M.L., Vauthey J.N., Dindo D., Schulick R.D., de Santibañes E., Pekolj J., Slankamenac K., Hamel C. (2009). The Clavien-Dindo classification of surgical complications: Five-year experience. Ann. Surg..

[17] Winstanley J., Ahmed S., Courtney M., Sam M., Mahawar K. (2021). One anastomosis gastric bypass in patients with gastroesophageal reflux disease and/or hiatus hernia. Obes. Surg..

[18] Chevallier J.M., Arman G.A., Guenzi M., Rau C., Bruzzi M., Beaupel N., Zinzindohoué F., Berger A. (2015). One thousand single anastomosis (omega loop) gastric bypasses to treat morbid obesity in a 7-year period: Outcomes show few complications and good efficacy. Obes. Surg..

[19] Liagre A., Debs T., Kasir R., Ledit A., Juglard G., du Rieu M.C., Lazzati A., Martini F., Petrucciani N. (2020). One anastomosis gastric bypass with a biliopancreatic limb of 150 cm: Weight loss, nutritional outcomes, endoscopic results, and quality of life at 8-year follow-up. Obes. Surg..

[20] Soprani A., Zulian V., Nedelcu M., Carandina S. (2022). One-Stage conversion of laparoscopic adjustable gastric banding to laparoscopic one anastomosis gastric bypass: A single-center experience on 1000 patients at 5 years of follow-up. Surg. Obes. Relat. Dis..

[21] Johari Y., Lim G., Wickremasinghe A., Yue H., Seah J., Ooi G., Playfair J., Laurie C., Beech P., Yap K. (2022). Pathophysiological Mechanisms of Gastro-esophageal Reflux After Sleeve Gastrectomy. Ann. Surg..

[22] Genco A., Soricelli E., Casella G., Maselli R., Castagneto-Gissey L., Di Lorenzo N., Basso N. (2017). Gastroesophageal reflux disease and Barrett’s esophagus after laparoscopic sleeve gastrectomy: A possible, underestimated long-term complication. Surg. Obes. Relat. Dis..

[23] Nehmeh W.A., Baratte C., Rives-Lange C., Martineau C., Boullenois H., Krivan S., Guillet V., Le Gall M., Cellier C., Carette C. (2021). Acid reflux is common in patients with gastroesophageal reflux disease after one-anastomosis gastric bypass. Obes. Surg..

[24] Gorodner V., Matucci A., Solé L., Figueredo R., Sánchez C., Caro L., Grigaites A. (2022). Does Roux-en-Y gastric bypass really cure gastroesophageal reflux disease? Analysis of Objective Data. J. Laparoendosc. Adv. Surg. Tech. A.

[25] Stenberg E., Ottosson J., Magnuson A., Szabo E., Wallén S., Näslund E., Thorell A., Näslund I. (2023). Long-Term Safety and Efficacy of Closure of Mesenteric Defect in Laparoscopic Gastric Bypass Surgery: A randomized Clinical Trial. JAMA Surg..

[26] Edholm D., Ottosson J., Sundbom M. (2016). Importance of pouch size in laparoscopic Roux-en-Y gastric bypass: A cohort study of 14,168 patients. Surg. Endosc..

[27] DeMeester S.R. (2020). Laparoscopic hernia repair and fundoplication for gastroesophageal reflux disease. Gastrointest. Endosc. Clin. N. Am..

